# The Coupling of Stride Length and Foot Strike in Running

**DOI:** 10.3389/fspor.2022.768801

**Published:** 2022-04-12

**Authors:** Missy Thompson, Kristine Hoffman, Lindsay Blythe, Rachel Hasler, Megan Longtain

**Affiliations:** ^1^Department of Health & Human Performance, Fort Lewis College, Durango, CO, United States; ^2^Department of Orthopedics, Denver Health Medical Center, Denver, CO, United States; ^3^Department of Orthopedics, University of Colorado School of Medicine, Aurora, CO, United States

**Keywords:** running, foot strike, stride length, biomechanics, kinematics, kinetics

## Abstract

Modifying stride length and/or foot strike in running results in mechanical alterations associated with injury risk. Stride length and foot strike have often been treated as independent factors that affect running mechanics, but there is evidence to suggest that they may be coupled. The purpose of this study was to determine if foot strike and stride length are coupled in running, and if so, can these variables be independently manipulated? Additionally, we sought to determine how independently and simultaneously manipulating stride length and foot strike influenced running kinematics and kinetics. Fifteen individuals ran over ground with stride lengths +/– 10 % of their preferred stride length while adopting both a fore/mid foot strike and rear foot strike pattern, as well as running with their self-selected stride length and foot strike when the opposite variable was controlled. Three-dimensional motion capture and force plate data were captured synchronously during the manipulated stride length x foot strike trials. The results indicate that foot strike and stride length are coupled, with shorter stride lengths being associated with a F/MFS and longer stride lengths being associated with a RFS pattern. Impact peak magnitude was primarily dependent on foot strike, with a F/MFS pattern reducing the magnitude of the impact peak force regardless of stride length. Peak vertical and horizontal ground reaction forces were found to be primarily dependent on stride length, with longer stride lengths resulting in increased vertical and horizontal ground reaction forces, regardless of foot strike. It is difficult, but possible, to independently manipulate stride length and foot strike. Clinicians should be aware of the coupled changes in stride length and foot strike.

## Introduction

Running is one of the most popular fitness and recreational activities worldwide. This is despite the fact that an estimated 37–56% of runners are injured annually, with 50 to 75% of these injuries being classified as overuse injuries that are due to the constant repetitive loading associated with running (van Mechelen, 1992). Running mechanics have been identified as being a main factor that can lead to overuse injury, with runners exhibiting abnormal movement patterns (kinematics) or excessive musculoskeletal loading (kinetics) being at a greater risk for injury (Hreljac, 2004; Davis and Futrell, 2016). In terms of kinematics, the magnitude and rate of foot pronation have been implicated as a contributing factor to overuse running injuries (Rolf, 1995). Key kinetic variables that have been linked to overuse injuries in runners include the magnitude of the impact forces, which results from the rapid collision of the foot with the ground, and the rate of impact loading (Hreljac, 2004).

Given the high incidence of overuse injuries in running considerable attention has been focused on understanding how kinematic adjustments can change running kinetics and the potential implications these alterations have on injury incidence (Davis and Futrell, 2016). In particular, the gait parameters of foot strike and stride length have received considerable attention in the scientific literature due to their implications for running related overuse injuries. While these variables are frequently discussed in isolation it is important to assess if gait alterations are linked and the implications that this may have on injury incidence.

Stride length has been identified as a modifiable gait parameter that results in mechanical alterations that are associated with reduced loading of biological tissues and, by extension, also may be associated with reduced injury risk. Decreased stride length has been associated with a reduction in impact forces and the active peak of the vertical GRF, as well as a decreased vertical loading rate of the GRF (Hobara et al., 2012). Additionally, decreasing stride length has been shown to reduce joint moments (Derrick et al., 1998; Heiderscheit et al., 2011; Thompson et al., 2014), impact accelerations (Smith et al., 1986; Hamill et al., 1995; Mercer et al., 2005), and leg stiffness (Farley and Gonzalez, 1996; Morin et al., 2007), which are factors that have been associated with increased risk of running-related overuse injuries. Clinically, evidence suggests that reducing stride length may reduce the incidence of tibial stress fractures (Ferber et al., 2002; Davis et al., 2004; Milner et al., 2006; Pohl et al., 2008) and plantar fasciitis (Pohl et al., 2009).

Similar to stride length, foot strike is a modifiable gait parameter with biomechanical implications associated with the incidence of running-related overuse injuries. When running with a rear foot strike (RFS) pattern the tibialis anterior is activated to decelerate plantar flexion as first the heel and then the mid and forefoot contact the ground. Correspondingly, a RFS pattern when running has been associated with increased pressures in the anterior compartment of the lower leg (Kirby and McDermott, 1983). Alternatively, when runners adopt a fore/mid foot strike (F/MFS) pattern the triceps surae muscles must be activated to slow dorsiflexion as first the forefoot contacts the ground followed by the rearfoot. Hence, F/MFS running has been associated with higher Achilles tendon strain and plantar flexor moments (Taunton et al., 2002). From a clinical perspective, adopting a F/MFS has been shown to improve chronic exertional compartment syndrome (Diebal et al., 2012). However, F/MFS running has also been associated with higher Achilles tendon strain and increased risk of Achilles tendinopathy (Jonsson et al., 2008; Azevedo et al., 2009).

Mechanical alterations and the corresponding clinical implications are evident with changes in both stride length and foot strike, but these variables have often been treated as independent factors in the literature. This is despite the fact that studies have reported data indicating that with a F/MFS pattern stride length is decreased (Altman and Davis, 2012a), suggesting that the variables of foot strike and stride length may be coupled. The purpose of this study was to determine if foot strike and stride length are coupled in running, and if so, can these variables be independently manipulated? Additionally, we sought to determine how independently and simultaneously manipulating stride length and foot strike influences running kinematics and kinetics. These findings have important implications because both variables have been identified as gait parameters that can potentially be modified to reduce running related overuse injuries. It is important to understand the mechanical implications of altering foot strike and stride length both individually and collectively to make informed treatment decisions.

## Materials and Methods

A total of 15 healthy active subjects (seven female, eight male); mass: 65.9 ± 9.8 kg; age: 24 ± 1.65 years volunteered to participate in this study. All participants were natural shod RFS runners, as defined by a foot strike angle >0^o^ in their preferred running gait (Altman and Davis, 2012a). All participants ran in their personal traditional running shoes, which had a minimum of a 15 mm forefoot stack height, 25 mm rearfoot stack height and 12 mm heel-toe drop. Subjects were required to perform a minimum of 30 min of physical activity at least 5 days a week and be free of musculoskeletal injury of the lower extremities or back in the past year. The Fort Lewis College Institutional Review Board approved the protocol for this study, and participants provided their written informed consent prior to participation.

### Experimental Protocol

The testing procedures are based on the protocol of Thompson et al. (2014). Participants took part in the following three laboratory testing sessions that were separated by at least 24 h.

- Session 1: Baseline measures of the participants' preferred running stride length, foot strike (verification of RFS) and velocity. In subsequent sessions foot strike and stride length were manipulated from these baseline measures, and velocity was controlled at the baseline value.
- Session 2: Foot strike and stride length were independently manipulated to examine the natural change in the corresponding gait parameter.
  ◦ Self-Selected Foot Strike (SSFS)—stride length manipulated to ±10% of baseline; foot strike was naturally selected.
  ◦ Self-Selected Stride Length (SSSL)—foot strike was manipulated to a F/MFS; stride length was naturally selected.
- Session 3—Foot strike and stride length were simultaneously manipulated. Stride length manipulated to ±10% of baseline and foot strike controlled to F/MFS and RFS (conditions: F/MFS +10%, F/MFS −10%, RFS +10%, RFS −10%).

Subjects began each session with 5–10 min of easy running to warm-up and habituate to the 20 m runway. During testing subjects ran in one direction down the runway and then jogged or walked back to the start. Participants were given 5–10 min at the start of each new condition to familiarize themselves with the parameters.

**Figure 1:**
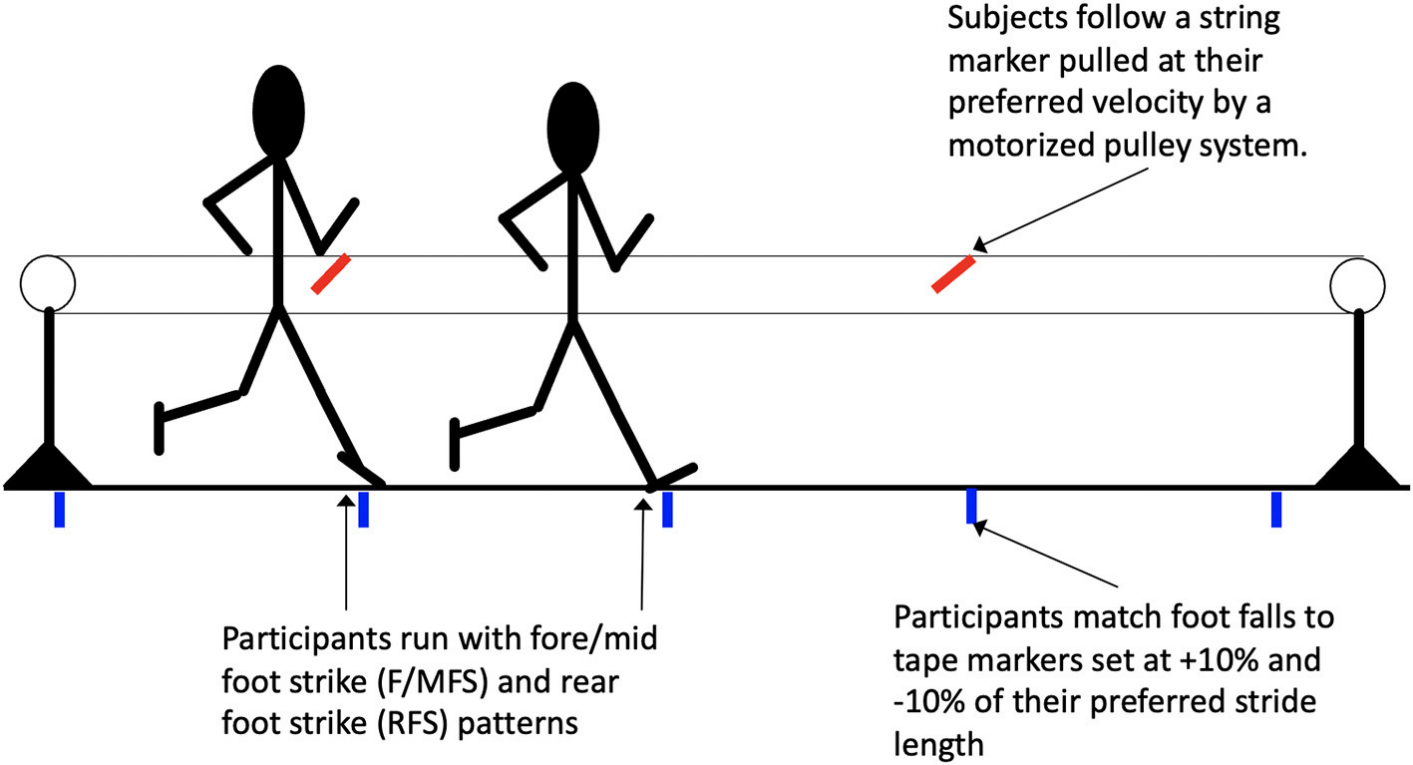
The experimental setup for the stride length (-10% and +10%) and foot strike (F/MFS and RFS) manipulation conditions.

In sessions 2 and 3 where stride length and foot strike were independently and simultaneously manipulated, velocity was controlled to that of the preferred condition by having subjects match their speed to a marker on a motor driven pulley system and stride length was controlled by having participants match foot falls to strips of tape placed along the runway (Figure 1). Trials in which velocity differed by >5% from the baseline condition or stride length differed by >5% across trials or from the desired stride length were excluded. For each stride length x foot strike condition 10 trials were completed and five strides (centered around the force plate) from each trial were used to calculate participant mean data for each condition. For the imposed stride length and foot strike conditions, foot strike, stride length and velocity were verified by 3D motion capture.

For all testing sessions kinematic data was captured via a motion analysis system as subjects ran across the 20 m runway. Participants wore retro-reflective markers which were affixed to the skin or tight-fitting clothing/shoes overlying specific anatomical landmarks based on the Modified Helen Hayes Marker set (Kadaba et al., 1990). Markers were placed bilaterally on the anterior and posterior superior iliac spines, lateral mid-thigh, lateral femoral epicondyle, lateral mid-shank, lateral malleolus, second metatarsal head and calcaneus. The same researcher was responsible for marker placements for all testing sessions.

### Preferred Stride Length, Foot Strike and Velocity

Session 1 was used to determine the participants' preferred stride length, foot strike (verification of RFS) and velocity while running in their personal traditional running shoes. Participants were instructed to run as they naturally would for a 1-h distance run. Stride length and running velocity were averaged from 10 strides recorded from each of 10 trials in which the participant naturally (i.e., did not adjust step length) contacted the force plate. Trials in which velocity or stride length differed by >5% were excluded. In subsequent testing sessions, velocity was controlled at the value measured in this session and stride length was manipulated based on the baseline value measured in this session.

### Self-Selected Foot Strike (SSFS)

In Session 2 participants completed the Self-selected Foot strike (SSFS) trials, which were randomized. For the SSFS trials participants ran with their stride length manipulated to ±10% of their baseline stride length and their foot strike was naturally selected. Coupling was identified when the manipulated stride length resulted in a consistent and significant change in foot strike across participants.

### Self-Selected Stride Length (SSSL)

In Session 2 participants also completed the Self-selected Stride Length (SSSL) trials, which were likewise randomized. For the SSSL trials participants ran with an imposed F/MFS and their stride length was naturally selected. Since all participants were natural RFS runners there was no imposed RFS condition. Coupling was identified when the manipulated foot strike resulted in a consistent and significant change in stride length across participants.

### Manipulated Stride Length and Foot Strike

In Session 3 participants ran with their stride length manipulated to ±10% of their preferred stride length while attempting to RFS and F/MFS (conditions: F/MFS +10%, F/MFS −10%, RFS +10%, RFS −10%). This was used to determine if the variables of foot strike or stride length could be uncoupled. At the end of the testing session participants completed questionnaire in which they were asked to rate the imposed foot strike x stride length trials in terms of level of difficulty.

### Kinematics

Three-dimensional marker position data were captured at 200 Hz with a Vicon Bonita system (Vicon, Oxford Metrics Ltd., UK) and this data was filtered using a Woltring filtering routine with a predicted mean square error of 4 mm^2^. Vicon Nexus software employing the PlugIn Gait model (Version 1.8.5, Vicon Motion Systems Ltd., Oxford, UK) was used to calculate three-dimensional kinematics of the ankle, knee and hip joint (Kadaba et al., 1990). Peak values for all joint angles and joint angles at ground contact were extracted for analysis.

Stride length was measured as the horizontal distance between the lowest position of the right and left heel markers. Running velocity was calculated as the horizontal displacement of each anterior superior iliac spine (ASIS) marker through the capture volume divided by the corresponding time. Running velocity was calculated for both the right and left ASIS markers and averaged. Foot strike was determined from foot strike angle (FSA) measured with the 3D motion capture system, which was calculated as the angle of the foot with respect to the ground in the sagittal plane. A FSA > 0^o^ was defined as RFS, and FSA <0^o^ was defined as F/MFS (Altman and Davis, 2012a).

### Kinetics

In addition to the kinematic data, GRF data were collected as subjects ran across the runway, which had an embedded force plate (AMTI, Waterton, MA). The three orthogonal components (vertical, horizontal and mediolateral) of the GRF data were recorded at 1,000 Hz from the force plate in synchrony with the motion capture data. The force plate data were low-pass filtered at 30 Hz using a second-order Butterworth filter, then down-sampled and combined with the motion capture data. Impact peak magnitude was determined as the first peak in the vertical GRF recording.

### Statistics

Differences in kinetic (vertical, horizontal and mediolateral GRF, and impact force) and kinematic parameters (3D peak joint angles and joint angles at ground contact) were analyzed using two-way repeated measure ANOVA tests. This allowed us to assess main effects of foot strike (SSFS, RFS, F/MFS) and stride length (SSSL, +10%, −10%), as well as the interaction of the foot strike and stride length conditions, on the kinematic and kinetic variables. For the SSSL analysis the difference in stride length between the SSFS (RFS) and F/MFS conditions was evaluated with a paired *t*-test. For the SSFS analysis a one-way repeated measure ANOVA test was used to compare foot strike angle between the SSSL, −10% and +10% conditions. We performed Newman–Keuls *post hoc* tests to ascertain the differences between conditions for the ANOVA tests. Significance was defined as p ≤ 0.05. All statistical tests were conducted in SPSS Version 23 (IBM, Armonk, NY). Coupling was defined as a significant change in foot strike when stride length was manipulated and vice versa. Effect sizes (d) were calculated to quantify the strength of any significant findings.

## Results

In the preferred condition participants ran with an average velocity of 3.43 ± 0.43 m/s and were able to run within 5% of this target velocity for the manipulated stride length and foot strike trials (Table 1). Motion capture data verified that the participants were able to adopt the imposed foot strike positions (F/MFS and RFS) at both the longer and shorter stride lengths (Table 1).

**Table 1:** Stride length normalized by leg length and velocity for the preferred and manipulated stride length and foot strike trials.

	**−10%**			**Preferred**	**+10%**		
	**F/MFS**	**RFS**	**SSFS**		**SSFS**	**F/MFS**	**RFS**
Stride length/leg length	2.33 (0.29)	2.34 (0.25)	2.38 (0.26)	2.62 (0.24)	2.88 (0.29)	2.84 (0.26)	2.89 (0.28)
Velocity (m/s)	3.27 (0.50)	3.36 (0.46)	3.19 (0.48)	3.34 (0.43)	3.25 (0.58)	3.41 (0.47)	3.45 (0.44)

*SSFS, self-selected foot strike—stride length was controlled and foot strike was naturally selected; F/MFS, fore/mid foot strike; RFS: rear foot strike; −10%, stride length manipulated to 10% shorter than stride length measured in the preferred condition; +10%, stride length manipulated to 10% longer than stride length measured in the preferred condition. For all manipulated stride length and foot strike trials velocity was controlled to that of the preferred condition*.

### Self-Selected Foot Strike (SSFS) and Self-Selected Stride Length (SSSL)

**Figure 2:**
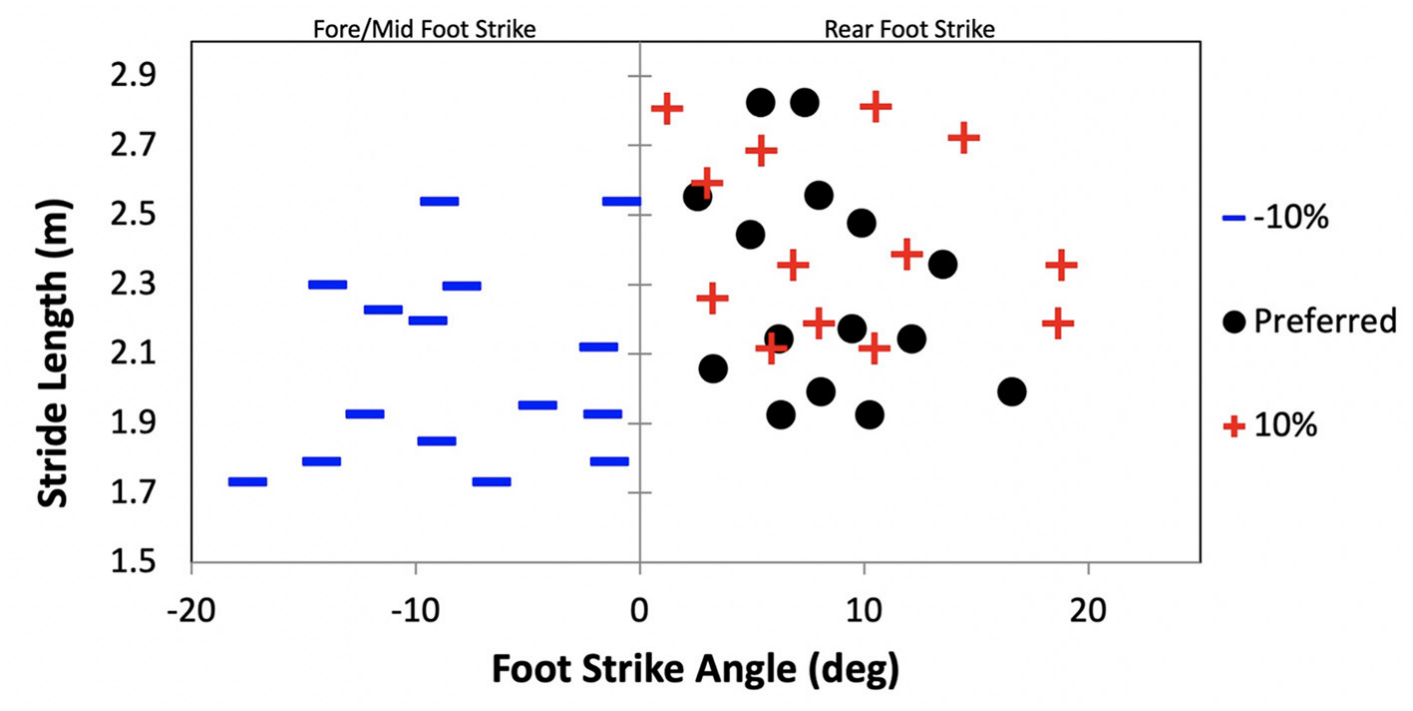
Foot strike angle (FSA) as a function of stride length for the preferred and self-selected foot strike (SSFS) −10% and +10% stride length conditions. RFS: FSA > 0^o^ and F/MFS: FSA < 0^o^.

**Figure 3:**
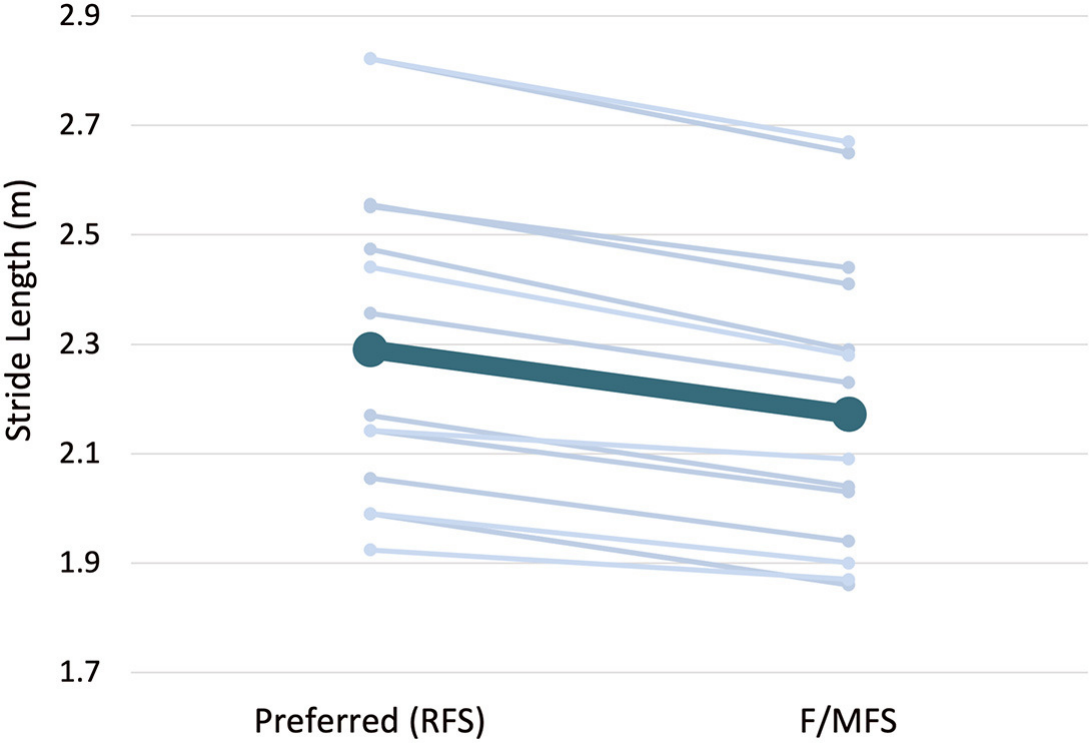
Preferred vs. self-selected stride length (SSSL) when adopting a F/MFS.

Findings from the SSFS and SSSL trials indicated that foot strike and stride length are coupled. The one-way ANOVA identified a significant difference in stride length between the SSFS conditions [*F*_(2,28)_ = 4.87, *p* = 0.02, *d* = 0.67]. For the SSFS conditions, all participants continued to RFS at the longer +10% stride length (FSA: 8.7 ± 5.4^o^, *p* = 0.23 vs. preferred) but adopted a F/MFS position with the shorter −10% stride length (FSA: −7.4 ± 5.3^o^, *p* = 0.01 vs. preferred; Figure 2). Likewise, there was a significant difference in stride length for the SSSL conditions, participants decreased stride length by an average of 5.3 ± 1.7 % from the preferred condition when using an imposed FFS (*p* = 0.02, *d* = 0.75, Figure 3).

Participants were able to adopt all conditions in which foot strike and stride length were simultaneously manipulated, indicating that foot strike and stride length can be uncoupled. However, participants noted increased difficulty with conditions which deviated from what was naturally selected (shortened stride length with RFS or lengthened stride length with F/MFS). The −10% RFS condition was unanimously ranked as the most difficult condition to maintain, followed by the +10% F/MFS condition.

### Kinematics

As expected, there were significant main effects of foot strike on ankle dorsiflexion angle at ground contact [*F*_(2,28)_ = 18.53, *p* < 0.001, *d* = 0.87], and foot strike angle at ground contact [*F*_(2,28)_ = 15.08, *p* < 0.001, *d* = 0.91]. The F/MFS conditions resulted in negative foot strike angles (F/MFS −10%: −11.3_± 5.0^o^; F/MFS +10%: −6.8 ± 4.2^o^) and were significantly decreased from the preferred (8.3 ± 3.8^o^) and RFS conditions (RFS −10%: 5.6 ± 5.1^o^; RFS +10%: 9.3 ± 5.0^o^) (Table 2). Likewise, the F/MFS conditions resulted in a more plantarflexed ankle position (negative dorsiflexion angle) at ground contact (F/MFS −10%: −5.2 ± 4.8^o^; F/MFS +10%: −4.7 ± 3.9^o^), which differed significantly from the preferred (5.2 ± 4.7^o^) and RFS conditions (RFS −10%: 6.2 ± 4.3^o^; RFS +10%: 7.1 ± 5.3^o^) that required a more dorsiflexed position at ground contact (positive dorsiflexion angle). Additionally, the longer +10% stride length conditions resulted in significantly greater hip flexion angles at ground contact (at ground contact: SSFS +10% = 35.2 ± 10.8^o^, *p* = 0.03; FFS +10% = 34.8 ± 11.4^o^, *p* = 0.04; RFS +10% = 35.6 ± 10.7^o^, *p* = 0.02) and peak hip flexion angles (peak: SFS +10% = 43.6 ± 10.7^o^, *p* = 0.02; FFS +10% = 44.1 ± 10.2^o^, *p* = 0.01; and RFS +10% = 44.8 ± 11.0^o^, *p* = 0.01) in comparison to the preferred condition (at ground contact = 33.8 ± 11.0^o^, peak = 40.5 ± 8.7^o^) (Table 2).

**Table 2:** Mean (SD) values for lower extremity kinematics at ground contact and peak values for the preferred and manipulated stride length and foot strike trials.

		**−10%**			**Preferred**	**+10%**		
	**FFS**	**RFS**	**SSFS**		**SSFS**	**FFS**	**RFS**	
Foot strike angle (^o^)	Ground contact	−11.3 (5.01)^*^	5.62 (5.14)	−7.41 (5.25)^*^	8.29 (3.83)	8.71 (5.38)	−6.75 (4.22)^*^	9.30 (4.98)
Ankle dorsiflexion (^o^)	Ground contact	−5.2 (4.8)^*^	6.2 (4.3)	−5.5 (5.1)^*^	5.2 (4.7)	6.6 (4.9)	−4.7 (3.9)^*^	7.1 (5.3)
	Peak	28.7 (7.2)^*^	29.3 (6.7)	27.6 (9.2)^*^	30.1 (6.6)	29.5 (8.1)	28.7 (7.2)^*^	32.5 (7.4)
Ankle adduction (^o^)	Ground contact	1.6 (6.5)	0.9 (7.1)	1.2 (6.9)	1.1 (5.7)	1.3 (5.5)	1.5 (7.3)	1.1 (5.8)
	Peak	8.2 (4.9)	7.9 (5.3)	7.8 (5.1)	6.7 (5.2)	8.1 (5.5)	6.9 (6.4)	6.5 (7.5)
Ankle internal rotation (^o^)	Ground contact	**–**6.3 (10.9)	**–**7.3 (11.1)	**–**6.9 (9.7)	**–**8.1 (11.2)	**–**7.2 (10.8)	**–**6.8 (11.4)	**–**5.3 (10.2)
	Peak	4.3 (9.7)	3.8 (8.9)	4.1 (10.8)	5.1 (9.4)	4.3 (10.6)	4.8 (9.6)	5.3 (10.2)
Knee flexion (^o^)	Ground contact	6.7 (4.9)	4.5 (7.6)	4.8 (6.7)	6.9 (5.2)	5.3 (6.2)	6.4 (5.9)	7.1 (6.6)
	Peak	34.9 (5.6)	36.7 (6.8)	36.2 (5.8)	35.9 (6.1)	36.0 (6.4)	35.3 (6.0)	37.2 (7.1)
Knee varus (^o^)	Ground contact	7.2 (5.0)	6.8 (4.7)	5.4 (6.2)	7.0 (4.8)	5.7 (6.1)	5.9 (5.4)	6.3 (5.6)
	Peak	17.5 (10.0)	18.5 (11.7)	19.4 (11.8)	17.5 (9.9)	18.2 (12.8)	16.8 (10.9)	17.5 (12.1)
Knee internal rotation (^o^)	Ground contact	**–**28.2 (13.9)	**–**29.2 (15.9)	**–**28.9 (16.1)	**–**28.5 (14.1)	**–**27.2 (13.5)	**–**29.5 (12.4)	**–**28.6 (12.5)
	Peak	1.9 (6.7)	2.2 (5.7)	2.3 (7.0)	2.0 (6.3)	2.2 (8.3)	1.8 (9.5)	2.2 (7.3)
Hip flexion (^o^)	Ground contact	35.6 (9.7)	33.6 (10.2)	34.5 (11.1)	33.8 (11.0)	35.2 (10.8)^*^	34.8 (11.4)^*^	35.6 (10.7)^*^
	Peak	32.9 (12.1)	31.7 (11.9)	32.8 (9.9)	40.5 (8.7)	43.6 (10.7)^*^	44.1 (10.2)^*^	44.8 (11.0)^*^
Hip adduction (^o^)	Ground contact	5.7 (5.0)	6.1 (6.2)	6.2 (5.4)	6.0 (5.9)	6.2 (5.7)	5.8 (5.4)	5.6 (6.1)
	Peak	12.9 (10.1)	12.6 (9.1)	13.5 (9.0)	12.7 (9.6)	13.1 (9.3)	12.9 (10.3)	13.0 (10.2)
Hip internal rotation (^o^)	Ground contact	23.1 (19.2)	26.5 (18.7)	25.6 (18.2)	29.1 (16.9)	27.2 (17.3)	25.9 (17.8)	27.6 (16.7)
	Peak	30.1 (13.9)	32.1 (12.4)	33.7 (10.8)	35.1 (11.8)	34.1 (11.3)	31.7 (11.5)	31.8 (13.2)

*Self-selected foot strike (SSFS)—stride length was controlled and foot strike was naturally selected. SSFS, self-selected foot strike—stride length was controlled and foot strike was naturally selected; F/MFS, fore/mid foot strike; RFS, rear foot strike; −10%: stride length manipulated to 10% shorter than stride length measured in the preferred condition; +10%: stride length manipulated to 10% longer than stride length measured in the preferred condition*.

* *Indicates a significant difference from the preferred condition (p ≤ 0.05). Bold indicates statistically significant*.

### Kinetics

**Figure 4:**
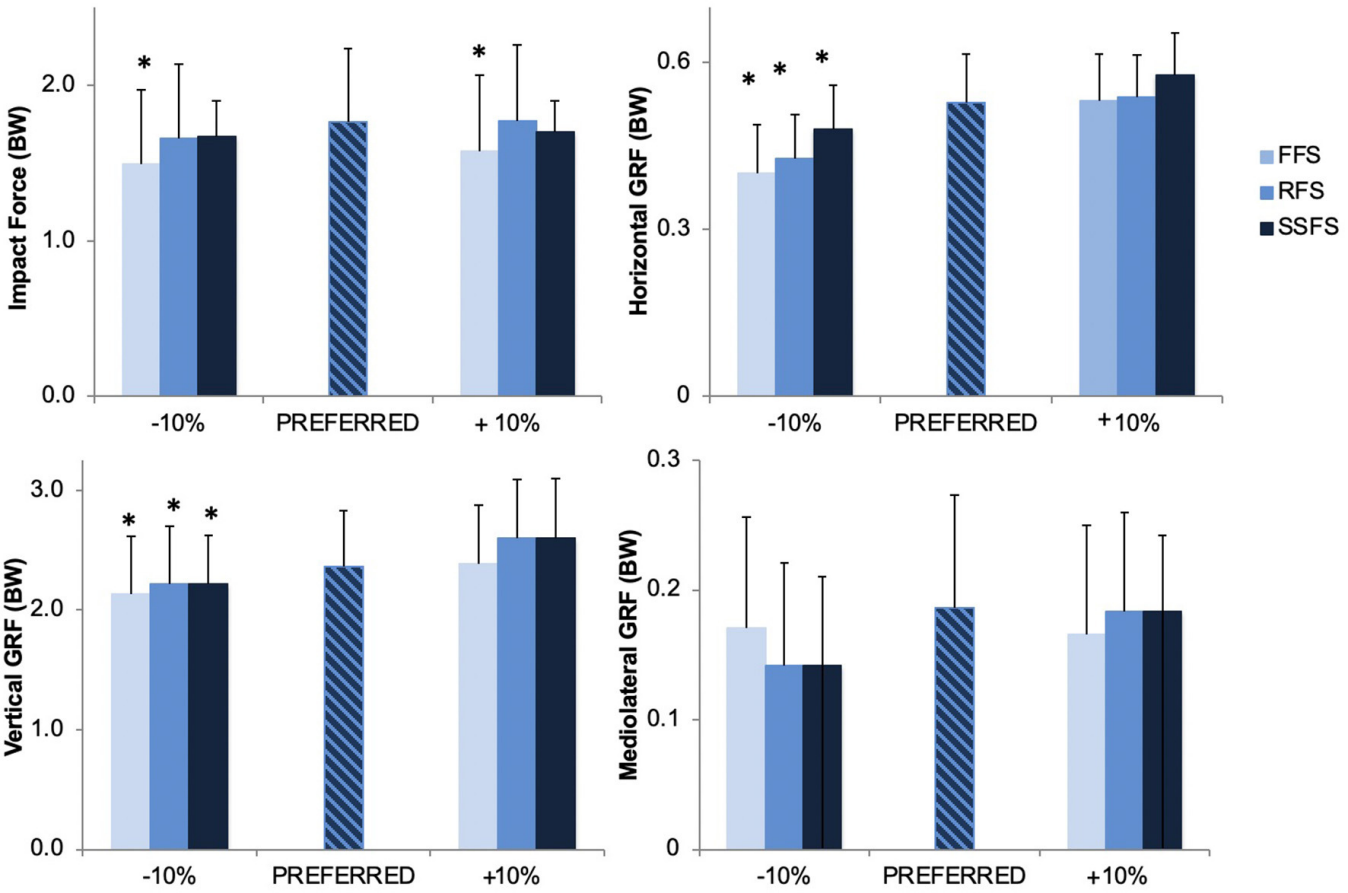
Ground reaction force components for running in the stride length and foot strike manipulated trials. *Indicates a significant difference from preferred condition, *p* ≤ 0.05.

There was a significant main effect of foot strike on impact force [*F*_(2,28)_ = 4.48, *p* = 0.026, *d* = 0.49], with both the FFS −10% (1.49 ± 0.19 BW) and FFS +10% (1.58 ± 0.20 BW) exhibiting significantly decreased impact forces in comparison to the preferred (1.76 ± 0.22 BW), RFS −10% (1.66 ± 0.23 BW) and RFS +10% (1.77 + 0.20 BW) conditions (Figure 4). Alternatively, there was a significant main effect of stride length on peak horizontal GRF [*F*_(2,28)_ = 3.57, *p* = 0.04] and vertical GRF [*F*_(2,28)_ = 4.01, *p* = 0.03, *d* = 0.42]. The shorter stride length conditions, FFS −10% (vertical = 2.13 ± 0.48 BW; horizontal = 0.40 ± 0.09 BW), RFS −10% (vertical = 2.22 ± 0.40 BW; horizontal = 0.43 ± 0.08 BW), and SSFS −10% (vertical = 2.22 ± 0.40 BW; horizontal = 0.48 ± 0.08 BW), showed significantly decreased peak vertical and horizontal GRFs relative to the preferred condition (vertical = 2.26 ± 0.47 BW; horizontal = 0.52 ± 0.09 BW), and the longer stride length conditions, FFS +10% (vertical = 2.38 ± 0.49 BW; horizontal = 0.53 ± 0.08 BW), RFS +10% (vertical = 2.60 ± 0.50 BW; horizontal = 0.54 ± 0.08 BW) and SSFS +10% (vertical = 2.60 ± 0.50 BW; horizontal = 0.58 ± 0.08 BW) conditions resulted in significantly increased peak horizontal and vertical GRFs relative to the preferred condition (Figure 4).

## Discussion

The purpose of this study was to examine the relationship between foot strike and stride length in running, and to determine if these variables were coupled and if so, could they be independently manipulated. Additionally, we sought to determine how independently and simultaneously manipulating stride length and foot strike influenced running kinematics and kinetics. Findings from the SSFS and SSSL trials indicate that foot strike and stride length are coupled, with a F/MFS being adopted in 100% of the shorter (-10%) stride length trials and a RFS pattern being maintained in 100% of the longer stride length (+10%) trials. This is consistent with the findings of Allen et al. (Allen et al., 2016) who increased stride length by means of a metronome and found a decrease in foot inclination at ground contact with an increase in step rate (reduced stride length). Expanding on these findings, in the present study participants were able to independently manipulate foot strike and stride length, but reported considerable difficulty in doing so, particularly when adopting foot strike patterns that deviated from what would normally be selected at a given stride length.

The finding of coupled stride length and foot strike has important implications for manipulating these variables as a potential means for modifying running mechanics in an attempt to reduce running related overuse injuries. Most prior research has independently evaluated the changes in stride length or foot strike with minimal consideration that these variables may be coupled. In one of the few studies to explore the coupling of foot strike and stride length, Baggaley et al. (2017) examined gait modifications designed to reduce the average vertical loading rate and also the secondary gait changes stemming from these modifications. Adopting a forefoot strike or shortened step length reduced vertical loading rate and eccentric work at the knee, but the forefoot strike also increased eccentric work at the ankle. This led the authors to conclude that forefoot strike may result in potentially injurious secondary affects that may not be observed when alternatively using a shortened step length. Hence, clinicians can utilize decreasing stride length and/or adopting a F/MFS as a tool to reduce loading at the knee, but it is contraindicated for individuals with Achilles tendon pathology to run with a F/MFS.

In terms of stride length, previous research has shown that decreasing stride length, which may have resulted in a F/MFS pattern, decreases the impact peak (Hobara et al., 2012) and active peak magnitude of the vertical GRF (Morin et al., 2007; Heiderscheit et al., 2011; Thompson et al., 2014), as well as decreasing the vertical loading rate of the GRF (Hobara et al., 2012). Additionally research examining alterations in foot strike has shown that RFS pattern results in increased vertical loading rates (Almeida et al., 2015; Rice et al., 2016). In the present study we found that the impact peak magnitude was primarily dependent on foot strike, with a F/MFS pattern reducing the magnitude of the impact peak force regardless of stride length. Alternatively, peak vertical and horizontal GRFs were found to be primarily dependent on stride length, with longer stride lengths resulting in increased vertical and horizontal GRFs, regardless of stride length. Clinically, reducing stride length can be used to reduce tibiofemoral contact forces (Willy et al., 2016) and can decrease the probability of tibial stress fractures (Edwards et al., 2009). However, the corresponding shift to a F/MFS when decreasing stride length makes this approach ill-advised in individuals with Achilles tendon pathology or other pathologies of the ankle.

There are several mechanical benefits of both reduced stride length and a F/MFS pattern that have important implications in terms of running-related overuse injuries. These benefits largely stem from reduced impact loading (Hreljac, 2004), with the F/MFS pattern reducing impact loading and the corresponding reduced stride length reducing peak vertical and horizontal GRFs. In particular the reduced stride length and F/MFS combination has the potential to decrease the incidence of tibial stress fractures (Ferber et al., 2002; Davis et al., 2004; Milner et al., 2006; Pohl et al., 2008) plantar fasciitis (Pohl et al., 2009), patellofemoral pain and chronic exertional compartment syndrome. However, it should be noted that reducing stride length may have performance implications as individuals tend to choose a stride length that is most economical (Hunter and Smith, 2007; Hunter et al., 2017). Additionally, a F/MFS pattern increases loading at the ankle joint (Rooney and Derrick, 2013) and Achilles tendon loading (Rice and Patel, 2017), making it counter indicated in individuals with pathologies of the ankle or Achilles tendon. Further, running in minimalist footwear, in which individuals typically adopt a F/MFS, has been associated with meta-tarsal stress fractures (Giuliani et al., 2011), and the development of foot bone marrow edema, a marker of added stress to the foot (Ridge et al., 2013). It remains unclear if these injuries are due to a lack of cushioning under the foot or from the F/MFS pattern, but clinicians should be aware of the potential for increased risk of injury and avoid recommending adoption of a F/MFS in individuals with foot pathologies.

A key reason that foot strike and stride length have received considerable attention in the running literature is the changes in these parameters that occur with different types of footwear. Specifically, running barefoot and in some minimalist shoes typically leads traditionally shod RFS runners to reduce stride length and adopt a F/MFS pattern (Altman and Davis, 2012b). Thus, these footwear modifications may act as a trigger to achieve the modifications of reduced stride length and a F/MFS pattern. It is important to note that these gait modifications are not observed in natural shod F/MFS runners (Thompson et al., 2015). Additionally, it is important to consider that in the present study all runners utilized traditional running shoes with a heel—toe drop that induces a plantar flexed position.

The present study is limited in that only RFS runners were studied. Previous research has shown that natural F/MFS exhibit different responses to running shoes (Thompson et al., 2015), and hence it is likely that natural F/MFS runners may respond differently to imposed changes in foot strike and stride length. Future research should examine the coupling of foot strike and stride length in F/MFS runners.

In conclusion, the results presented here indicate that foot strike and stride length are coupled, i.e., changing stride length leads to a corresponding change in foot strike and vice versa. It is possible for individuals to uncouple these parameters; however, it is done with difficulty. When suggesting alterations in stride length or foot strike, clinicians should be aware of the associated changes in the coupled variable.

## Data Availability Statement

The raw data supporting the conclusions of this article will be made available by the authors, without undue reservation.

## Ethics Statement

The studies involving human participants were reviewed and approved by Fort Lewis College Institutional Review Board. The patients/participants provided their written informed consent to participate in this study.

## Author Contributions

All authors listed have made a substantial, direct, and intellectual contribution to the work and approved it for publication.

## Conflict of Interest

The authors declare that the research was conducted in the absence of any commercial or financial relationships that could be construed as a potential conflict of interest.

## Publisher's Note

All claims expressed in this article are solely those of the authors and do not necessarily represent those of their affiliated organizations, or those of the publisher, the editors and the reviewers. Any product that may be evaluated in this article, or claim that may be made by its manufacturer, is not guaranteed or endorsed by the publisher.
